# Trial Publication after Registration in ClinicalTrials.Gov: A Cross-Sectional Analysis

**DOI:** 10.1371/journal.pmed.1000144

**Published:** 2009-09-08

**Authors:** Joseph S. Ross, Gregory K. Mulvey, Elizabeth M. Hines, Steven E. Nissen, Harlan M. Krumholz

**Affiliations:** 1 Department of Geriatrics and Adult Development, Mount Sinai School of Medicine, New York, New York, United States of America; 2 HSR&D Research Enhancement Award Program and Geriatrics Research, Education, and Clinical Center, James J. Peters VA Medical Center, Bronx, New York, United States of America; 3 Center for Outcomes Research and Evaluation, Yale-New Haven Hospital, New Haven, Connecticut, United States of America; 4 Amherst College, Amherst, Massachusetts, United States of America; 5 Department of Cardiovascular Medicine, Cleveland Clinic, Cleveland, Ohio, United States of America; 6 Robert Wood Johnson Clinical Scholars Program and Section of Cardiolovascular Medicine, Department of Medicine, Yale University School of Medicine, New Haven, Connecticut, United States of America; 7 Section of Health Policy and Administration, Yale University School of Epidemiology and Public Health, New Haven, Connecticut, United States of America; University of California San Francisco, United States of America

## Abstract

**Background:**

ClinicalTrials.gov is a publicly accessible, Internet-based registry of clinical trials managed by the US National Library of Medicine that has the potential to address selective trial publication. Our objectives were to examine completeness of registration within ClinicalTrials.gov and to determine the extent and correlates of selective publication.

**Methods and Findings:**

We examined reporting of registration information among a cross-section of trials that had been registered at ClinicalTrials.gov after December 31, 1999 and updated as having been completed by June 8, 2007, excluding phase I trials. We then determined publication status among a random 10% subsample by searching MEDLINE using a systematic protocol, after excluding trials completed after December 31, 2005 to allow at least 2 y for publication following completion. Among the full sample of completed trials (*n = *7,515), nearly 100% reported all data elements mandated by ClinicalTrials.gov, such as intervention and sponsorship. Optional data element reporting varied, with 53% reporting trial end date, 66% reporting primary outcome, and 87% reporting trial start date. Among the 10% subsample, less than half (311 of 677, 46%) of trials were published, among which 96 (31%) provided a citation within ClinicalTrials.gov of a publication describing trial results. Trials primarily sponsored by industry (40%, 144 of 357) were less likely to be published when compared with nonindustry/nongovernment sponsored trials (56%, 110 of 198; *p*<0.001), but there was no significant difference when compared with government sponsored trials (47%, 57 of 122; *p* = 0.22). Among trials that reported an end date, 75 of 123 (61%) completed prior to 2004, 50 of 96 (52%) completed during 2004, and 62 of 149 (42%) completed during 2005 were published (*p* = 0.006).

**Conclusions:**

Reporting of optional data elements varied and publication rates among completed trials registered within ClinicalTrials.gov were low. Without greater attention to reporting of all data elements, the potential for ClinicalTrials.gov to address selective publication of clinical trials will be limited.

*Please see later in the article for the Editors' Summary*

## Abstract 2

Joseph Ross and colleagues examine publication rates of clinical trials and find low rates of publication even following registration in Clinicaltrials.gov.

## Editors' Summary

**Background:**

People assume that whenever they are ill, health care professionals will make sure they get the best available treatment. But how do clinicians know which treatment is most appropriate? In the past, clinicians used their own experience to make treatment decisions. Nowadays, they rely on evidence-based medicine—the systematic review and appraisal of the results of clinical trials, studies that investigate the efficacy and safety of medical interventions in people. However, evidence-based medicine can only be effective if all the results from clinical trials are published promptly in medical journals. Unfortunately, the results of trials in which a new drug did not perform better than existing drugs or in which it had unwanted side effects often remain unpublished or only appear in the public domain many years after the drug has been approved for clinical use by the US Food and Drug Administration (FDA) and other governmental bodies.

**Why Was This Study Done?:**

The extent of this “selective” publication, which can impair evidence-based clinical practice, remains unclear but is thought to be substantial. In this study, the researchers investigate the problem of selective publication by systematically examining the extent of publication of the results of trials registered in ClinicalTrials.gov, a Web-based registry of US and international clinical trials. ClinicalTrials.gov was established in 2000 by the US National Library of Medicine in response to the 1997 FDA Modernization Act. This act required preregistration of all trials of new drugs to provide the public with information about trials in which they might be able to participate. Mandatory data elements for registration in ClinicalTrials.gov initially included the trial's title, the condition studied in the trial, the trial design, and the intervention studied. In September 2007, the FDA Amendments Act expanded the mandatory requirements for registration in ClinicalTrials.gov by making it necessary, for example, to report the trial start date and to report primary and secondary outcomes (the effect of the intervention on predefined clinical measurements) in the registry within 2 years of trial completion.

**What Did the Researchers Do and Find?:**

The researchers identified 7,515 trials that were registered within ClinicalTrials.gov after December 31, 1999 (excluding phase I, safety trials), and whose record indicated trial completion by June 8, 2007. Most of these trials reported all the mandatory data elements that were required by ClinicalTrials.gov before the FDA Amendments Act but reporting of optional data elements was less complete. For example, only two-thirds of the trials reported their primary outcome. Next, the researchers randomly selected 10% of the trials and, after excluding trials whose completion date was after December 31, 2005 (to allow at least two years for publication), determined the publication status of this subsample by systematically searching MEDLINE (an online database of articles published in selected medical and scientific journals). Fewer than half of the trials in the subsample had been published, and the citation for only a third of these publications had been entered into ClinicalTrials.gov. Only 40% of industry-sponsored trials had been published compared to 56% of nonindustry/nongovernment-sponsored trials, a difference that is unlikely to have occurred by chance. Finally, 61% of trials with a completion date before 2004 had been published, but only 42% of trials completed during 2005 had been published.

**What Do These Findings Mean?:**

These findings indicate that, over the period studied, critical trial information was not included in the ClinicalTrials.gov registry. The FDA Amendments Act should remedy some of these shortcomings but only if the accuracy and completeness of the information in ClinicalTrials.gov is carefully monitored. These findings also reveal that registration in ClinicalTrials.gov does not guarantee that trial results will appear in a timely manner in the scientific literature. However, they do not address the reasons for selective publication (which may be, in part, because it is harder to publish negative results than positive results), and they are potentially limited by the methods used to discover whether trial results had been published. Nevertheless, these findings suggest that the FDA, trial sponsors, and the scientific community all need to make a firm commitment to minimize the selective publication of trial results to ensure that patients and clinicians have access to the information they need to make fully informed treatment decisions.

**Additional Information:**

Please access these Web sites via the online version of this summary at http://dx.doi.org/10.1371/journal.pmed.1000144.

- *PLoS Medicine* recently published two related articles on selected publication by Ida Sim and colleagues and by Lisa Bero and colleagues and an editorial discussing the FDA Amendments Act
- ClinicalTrials.gov provides information about the US National Institutes of Health clinical trial registry, including background information about clinical trials, and a fact sheet detailing the requirements of the FDA Amendments Act 2007 for trial registration
- The US Food and Drug Administration provides further information about drug approval in the US for consumers and health care professionals

## Introduction

Selective clinical trial publication, including nonpublication and delayed publication of completed trials, distorts the evidence available in the medical literature, compromising systematic reviews and meta-analyses, impairing evidence-based clinical practice, and undermining guideline recommendations. The extent of selective publication is not known, but previous studies have estimated between 25%–50% of supporting trials for US Food and Drug Administration (FDA)-approved drugs remained unpublished more than 5 y after approval [1],[2]. Similarly unpublished clinical trials of rosiglitazone [3] identified from a company-maintained website and of erythropoiesis-stimulating agents [4] and antidepressants [5] found among data submitted to the FDA revealed important efficacy and safety information to be missing from the medical literature. Such selective publication is raising questions about the frequency with which trials are unpublished and how best to ensure timely public and professional access to all trial results.

Section 113 of the 1997 FDA Modernization Act was enacted in the United States over 10 y ago to provide the public access to information about ongoing clinical trials in which they may be able to participate. The act required the creation of a public resource for information on studies of drugs, including biological drug products, which treat “serious or life-threatening” diseases and conditions conducted under the FDA's investigational new drug regulations, mandating the collection of specific descriptive information pertaining to each clinical trial. In response, the US National Library of Medicine (NLM) established the Web-based registry ClinicalTrials.gov in 2000, on behalf of the US National Institutes of Health (NIH), providing what was intended to be a publicly available, easily searchable, on-line source of information for all registered trials, including trials located domestically within the US and internationally. This registry has the potential to address selective publication by publicly cataloguing clinical trials and promoting trial transparency and accountability. In 2004, the International Committee of Medical Journal Editors (ICMJE) announced that any clinical trial must be registered by September 2005 in a public clinical trials registry that satisfied several specifications to be considered for publication in one of its journals; at that time, only ClinicalTrials.gov met the specifications put forth by the editors [6]. Between May and October 2005, the number of trials registered within ClinicalTrials.gov increased by 73% [7].

Despite these efforts, problems with the Web-based registry have been identified. An audit in 2005 of ClinicalTrials.gov by investigators at the NLM found one-quarter of registered trials did not describe the primary outcome defined within the study, and many of those that did lacked specific information about its timing and measurement [7]. No published study, however, has systematically examined the frequency and timeliness with which results of trials registered within ClinicalTrials.gov are published in the medical literature, a measure of how well ClinicalTrials.gov might be addressing selective publication.

The FDA Amendments Act (FDAAA), enacted in September 2007 in the US, included new initiatives to use ClinicalTrials.gov to further address selective publication. The legislation requires the sponsors of all drug, biologic, and device trials to register their studies, at inception, in the publicly available ClinicalTrials.gov database (with the exception of phase I clinical trials). Moreover, the registry must be updated to include information on participants and trial results for approved drugs and devices within 12 mo of study completion (24 mo if the studied drug is currently under review at the FDA); specifically, investigators must report the primary and principal secondary outcome results to ClinicalTrials.gov for publication within the registry. As details of legislation implementation remain under negotiation, there is a need for information about currently registered studies and the extent of selective publication. While this information is clearly relevant to policy-makers, it also has profound implications in terms of the evidence made available for clinicians, researchers, and patients. Accordingly, our objectives were to determine the completeness of registrations within ClinicalTrials.gov and determine the extent and correlates of selective publication.

## Methods

### Overview

ClinicalTrials.gov uses a Web-based system to facilitate clinical trial registration by any sponsor, principal investigator, or other person or organization with primary responsibility for the trial. Trials are defined by ClinicalTrials.gov as “… Research studies in human volunteers to answer specific health questions. Interventional trials determine whether experimental treatments or new ways of using known therapies are safe and effective under controlled environments. Observational trials address health issues in large groups of people or populations in natural settings” [8]. ClinicalTrials.gov serves as a registry for trials located both in the US and internationally and multisite clinical trials that are conducted using the same protocol are considered one trial in the registry. ClinicalTrials.gov includes mandatory and optional data elements (Table S1). Trials cannot be registered without completion of all mandatory data elements, approval by a human subject review board (or equivalent), and conformity to the regulations of the appropriate national health authorities. Additional information about the registry is available from the NLM [9].

### Study Sample and Variables for Completeness Analysis

**Figure 1:**
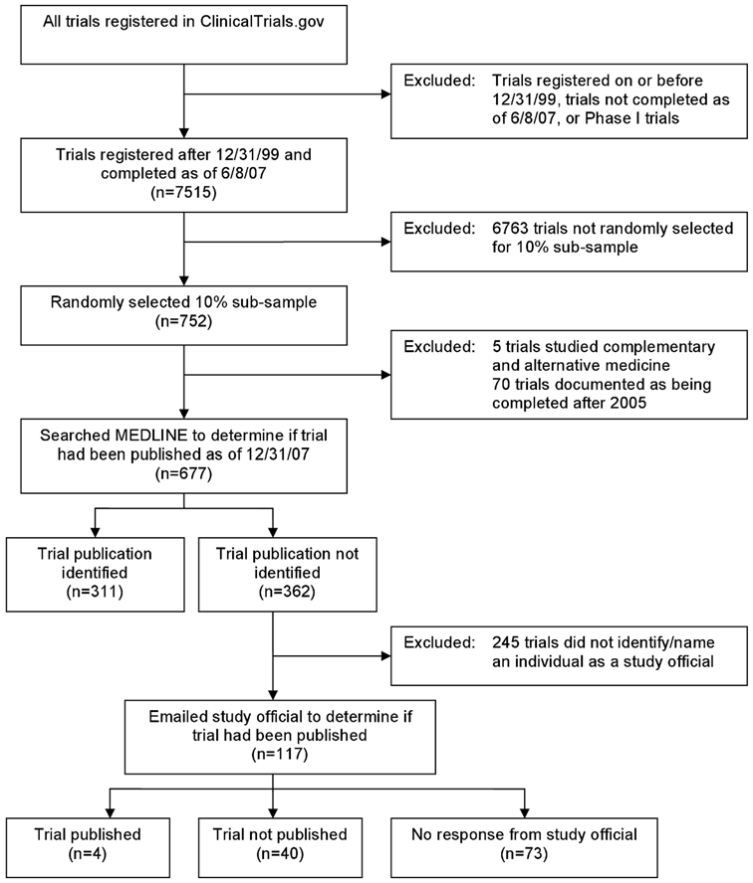
ClinicalTrials.gov trial inclusion flow chart.

Among more than 42,000 trials registered within ClinicalTrials.gov as of June 2007, we limited our study to clinical trials that were registered after December 31, 1999 and whose registry was updated to notify ClinicalTrials.gov that the trial had been completed as of June 8, 2007, excluding phase I trials (Figure 1). A completed trial is defined by ClinicalTrials.gov as a study that has concluded and participants are no longer being examined or treated (i.e., last patient's last visit has occurred) [10]. We obtained information on these trials through a request to the NLM, requesting the following mandatory data elements for each trial: identification number, title, primary sponsor, study official, design, type, phase (if interventional), intervention, condition, and population studied, along with the following optional data elements: enrollment, trial start and end dates, primary and secondary outcome measures, and publication. These data elements were requested (as opposed to all data elements) because we determined that each was relevant for identifying publications of registered trials and for examining associations between publication and several trial characteristics (e.g., sponsorship, condition studied). Data from the NLM were provided in a spreadsheet. Categorizations of data elements are made by study investigators/sponsors as part of trial registration. For instance, primary condition studied was assigned to one of 23 categories, primary study sponsor to one of six. We further categorized sponsor into three groups: government (US or non-US), industry, or nongovernment/nonindustry, which included universities, organizations, foundations, and clinical research networks. We used study design to categorize study purpose as efficacy only, efficacy and safety, safety only, or indeterminate. For instance, a study design of “Treatment/Randomized/Placebo Control/Safety/Efficacy Study” was categorized as efficacy and safety, whereas a study design of “Treatment/Randomized/Placebo Control/Safety Study” was categorized as safety only.

### Study Sample and Variables for Publication Analysis

From our full sample of completed trials, we created a 10% subsample by assigning each trial a random number and selecting those first in the randomization sequence to determine their publication status. For this analysis, we also excluded trials with a registered end date after December 31, 2005, in order to provide at least a 2 y period within which trials might be published, consistent with FDAAA legislation. For those trials that did not provide an end date within ClinicalTrials.gov, but did provide a start date, we excluded trials for which data collection started after June 30, 2005 for the same reason. We also excluded trials that studied complementary and alternative medicine, such as acupuncture or ginseng, as they were not our focus and we were concerned that these trials were not appropriate for comparison with “traditional” biomedical trials.

For all trials within the 10% subsample, we determined the following: publication status, study type, randomized design, and study location. Two of three authors (JSR, GKM, EMH) independently determined the publication status using a search protocol. All searches began by first examining the “publication” field within ClinicalTrials.gov to determine if trial investigators provided a citation of an article that described trial results, as this field is used to display citations of trial results or other relevant research, as provided by investigators. If no citation was provided, we then searched MEDLINE using the ClinicalTrials.gov identification number. If no publication was identified, MEDLINE was again searched using the intervention, condition studied, and the principal investigator (when provided in response to the “study official” field). The articles identified through the search were matched to the corresponding trial (when possible) using the following information from ClinicalTrials.gov: description, location, enrollment, start and end dates, and primary and secondary outcome measures. Any differences were resolved by consensus. Finally, if no publication was identified, we attempted to contact the study official identified within ClinicalTrials.gov to determine if the trial had been published, limiting our attempts to a maximum of three electronic mail messages.

Once a publication was identified for a registered trial, we determined whether the primary outcome described in the manuscript was the same as the primary outcome described within ClinicalTrials.gov.

### Statistical Analysis

We conducted a descriptive analysis, describing data quality, including completeness of reporting for each data element, and summarizing the characteristics of our sample by primary sponsor, type, purpose, phase, location, and condition and population studied. We then used Chi-square tests to examine the association between these trial characteristics and publication status. Because our 10% subsample excluded trials with a registered end date after December 31, 2005 in order to provide at least 2 y for publication, but included trials that did not report an end date, we examined the robustness of our results in two ways. First, we tested the interaction between end date reporting (yes/no) and each trial characteristic whose association with publication status was examined (i.e., sponsor, study location). No trial characteristic variable interacted significantly with end date reporting. Second, we repeated our analyses using a time-to-publication approach among only those trials that reported a trial end date. These analyses confirmed our main findings. Therefore, only the results from the full 10% subsample analyses are presented. Statistical analysis was performed using JMP 7.0.1 and SAS 9.1 (both from SAS Institute, Inc.). All statistical tests were two-tailed, using a type I error rate of 0.005 to account for multiple comparisons. Yale University Human Investigation Committee approval was obtained prior to the study.

## Results

### Completeness Analyses

There were 7,515 registered clinical trials in our analysis. Nearly 100% of records provided all mandatory data elements: title, sponsor, condition studied, design, type, phase, and intervention and population studied. Study official, which is also a mandatory data element, was also provided by 100% of records, with varying degrees of specificity: 63% provided the principal investigator contact name, whereas the others provided another study contact, such as the name of an institution, company, or facility. Reporting of optional data elements varied; 82% provided enrollment, 87% start date, 53% end date, 66% primary outcome measure, and 56% secondary outcome measure(s).

Nearly half of trials (44%) were primarily sponsored by industry, and cancer was the most common condition studied (13%, Table 1). Few studies described trials that were conducted only for safety (4%), although most were described as being conducted for safety and efficacy (44%). Among interventional trials, 34% were described as phase III or phase II/III, 31% were phase II or phase I/II, and 18% were phase IV. More than one-quarter of trials enrolled children (28%).

**Table 1:** Characteristics of completed trials registered in the ClinicalTrials.gov database after December 31, 1999 (excluding phase I trials).

Trial Characteristic		*n* (%)^a^ (*n* = 7,515)
Sponsor	Industry	3,330 (44%)
	Nongovernment/Nonindustry	2,824 (38%)
	Government (US and non-US)	1,361 (18%)
Condition studied	Cancers and other neoplasms	973 (13)
	Behaviors and mental disorders	727 (10)
	Heart and blood diseases	727 (10)
	Nutritional and metabolic diseases	687 (9)
	Conditions of the urinary tract and sexual organs, and pregnancy	522 (7)
	Viral diseases	467 (6)
	Nervous system diseases	461 (6)
	Respiratory tract (lung and bronchial) diseases	363 (5)
	Bacterial and fungal diseases	260 (4)
	Other	2,328 (31)
Study purpose	Safety and efficacy	3,304 (44)
	Efficacy only	1,737 (23)
	Safety only	297 (4)
	Indeterminate	2,177 (29)
Study type	Interventional	6,674 (89)
	Observational	841 (11)
Study phase^b^	Phase I/phase II or phase II	2,059 (31)
	Phase II/phase III or phase III	2,295 (34)
	Phase IV	1,110 (17)
	Not applicable	1,209 (18)
Population studied	Included adults	6,850 (91)
	Included older adults (age ≥65 y)	5,468 (73)
	Included children	2,076 (28)

a Proportions may not sum to 100 because of rounding.

b Data element is only required to be reported for interventional studies (*n* = 6,674).

### Publication Analyses

The random 10% subsample included 752 trials and 75 met at least one of our additional exclusion criteria (Figure 1). Among 677 included trials, 311 (46%) were published and indexed within MEDLINE (Table 2). Of these, 215 (69%) did not provide a citation within ClinicalTrials.gov of an article that described trial results, whereas 96 (31%) did.

**Table 2:** Publication rates stratified by trial characteristics among a 10% random subsample of completed trials registered in the ClinicalTrials.gov database.

Trial Characteristic	*n* of Trials Published/Total *n* (%)	*p*-Value
Overall	311/677 (46)	—
Sponsor	—	0.003*
Industry	144/357 (40)	—
Nongovernment/nonindustry	110/198 (56)	—
Government agencies (US and non-US)	57/122 (47)	—
NIH (US)	30/74 (41)	—
US federal agency, excluding NIH	19/34 (56)	—
Government, excluding US federal	8/14 (57)	—
Condition studied	—	0.23
Cancers and other neoplasms	43/102 (42)	—
Behaviors and mental disorders	33/62 (53)	—
Heart and blood diseases	22/46 (48)	—
Nutritional and metabolic diseases	24/52 (46)	—
Conditions of the urinary tract and sexual organs, and pregnancy	24/43 (56)	—
Viral diseases	18/54 (33)	—
Nervous system diseases	21/44 (48)	—
Respiratory tract (lung and bronchial) diseases	13/40 (33)	—
Bacterial and fungal diseases	22/40 (55)	—
Other	91/194 (47)	—
Study purpose	—	0.23
Safety and efficacy	139/307 (45)	—
Efficacy only	71/133 (53)	—
Safety only	12/31 (39)	—
Indeterminate	89/206 (43)	—
Study type	—	<0.001**
Comparison: intervention with placebo	140/248 (56)	—
Randomization?	—	0.75
Yes	124/221 (56)	—
No	16/27 (59)	—
Comparison: intervention with other active agent	96/224 (43)	—
Randomization?	—	0.17
Yes	85/205 (41)	—
No	11/19 (58)	—
No comparison: intervention alone	47/138 (34)	—
Observational (no intervention)	28/67 (42)	—
Study phase^a^	—	0.008
Phase I/phase II or phase II	66/184 (36)	—
Phase II/phase III or phase III	114/235 (49)	—
Phase IV	57/109 (52)	—
Trial size^b^	—	0.37
≥160 Participants	131/283 (46)	—
<160 Participants	120/282 (43)	—
Population studied	—	—
Included older adults (≥65 y)	230/499 (46)	0.89^c^
Included children (<18 y)	82/194 (42)	0.22^|d^
Study location	—	0.30
US/Canada only	148/339 (44)	—
US/Canada and international	20/43 (47)	—
International only	87/192 (45)	—
Not provided	56/103 (54)	—
Trial end date	—	0.005***
No end date provided	124/309 (40)	—
End date provided	187/368 (51)	—
Before January 1, 2004	75/123 (61)	—
Between January 1, 2004 and December 31, 2004 (inclusive)	50/96 (52)	—
Between January 1, 2005 and December 31, 2005 (inclusive)	62/149 (42)	—

*: *p*-Value for Pearson Chi-square testing the null hypothesis that publication rates among industry, government, and nonindustry/nongovernment sponsored trials are no different.

**: *p*-Value for Pearson Chi-square testing the null hypothesis that publication rates among trials that compared an intervention with placebo, trials that compared an intervention with another active agent, trials with no comparison, and observational studies are no different.

***: *p*-Value for Pearson Chi-square testing the null hypothesis that publication rates among trials with and without end dates provided are no different.

a Among interventional trials that reported a trial phase only.

b The median trial sample size was 160.

c In comparison with trials that did not include older adults.

d In comparison with trials that did not include children.

Among the 10% subsample of 677 trials, study end date was not reported for 309 (46%), although each had its registry updated within ClinicalTrials.gov to notify officials that the trial had been completed. Among 368 trials that provided an end date, 123 (33%) ended prior to 2004, 96 (26%) during 2004, and 149 (40%) during 2005. Trials primarily sponsored by industry, conducted only for safety, studied cancer, did not include children, and conducted in both US domestic and international sites were less likely to report an end date (*p*<0.005). Trials that reported a study end date were more likely to be published when compared with trials that did not (51% versus 40%; relative risk [RR] = 1.27, 95% confidence interval [CI] 1.07–1.50; *p* = 0.005). Among trials that reported an end date, 75 of 123 (61%) completed prior to 2004, 50 of 96 (52%) completed during 2004, and 62 of 149 (42%) completed during 2005 were published (*p* = 0.006).

Among the 10% subsample, industry was the primary sponsor of 357 (53%) trials, government 122 (18%), and nongovernment/nonindustry 198 (29%). Trials primarily sponsored by industry were less likely to be published when compared with nongovernment/nonindustry sponsored trials (40% versus 56%; RR = 0.73, 95% CI 0.61–0.87; *p*<0.001); there was no statistically significant difference in publication rates between industry and government primary sponsored trials (40% versus 47%; *p* = 0.22). Among government sponsored trials, only 42% of those primarily sponsored by the NIH were published (30 of 74). Among industry sponsors with ten or more trials in our subsample, publication rates varied widely: 13 of 14 (93%) trials primarily sponsored by Merck, seven of 11 (64%) by Amgen, and 15 of 24 (63%) by Johnson & Johnson were published, whereas only seven of 27 (26%) trials primarily sponsored by Novartis, nine of 34 (27%) by GlaxoSmithKline, and four of 14 (29%) by Sanofi-Aventis were published.

Among the 10% subsample, 248 (37%) trials compared an intervention with placebo (89% were randomized), 224 (33%) compared an intervention with another active agent (92% were randomized), 138 (20%) examined an intervention without a comparison group, and 67 (10%) were observational (no intervention). Trials comparing an intervention with placebo were more likely to be published when compared with other trial designs (56% versus 40%; RR = 1.42, 95% CI 1.21–1.66; *p*<0.001), and phase II trials were less likely to be published when compared with phase III or phase IV trials (36% versus 50%; RR = 0.72, 95% CI 0.58–0.90; *p* = 0.002). Other examined trial characteristics were not significantly associated with publication: condition or population studied, study purpose or location, or trial size.

Among 311 published trials, 198 (64%) reported a primary outcome within ClinicalTrials.gov, nearly all of which (97%) matched the primary outcome measure in the published manuscript. However, the data quality varied markedly, particularly its degree of specificity with regards to providing the time period after which the outcome will be studied and how the outcome will be measured. As an example, one trial reported the primary outcome “change from baseline to 6-mo in distal femur bone mineral density,” while another reported “bone mineral density.”

## Discussion

Our study demonstrates that the potential of ClinicalTrials.gov registry to address selective publication and better inform the public and professionals about the results from completed clinical trials is limited because critical information from trial registration, such as study contact, trial end date, and primary outcome, were not consistently reported. Moreover, publication rates among completed trials registered within ClinicalTrials.gov were low, even among trials with at least 4 y documented since study completion. Low publication rates were widespread among differing trial sponsors, conditions studied, study types, and locations. However, we also found significantly different publication rates among study types and primary sponsors, consistent with prior research [11]. Even when trials were found to be published, for the majority the citation was not available within ClinicalTrials.gov, which would have made it easy for the public and professionals to access results.

We expected that the trials we examined were likely to have been published in that they were recently completed after being registered at ClinicalTrials.gov within the past decade, ensuring that thet trial was in compliance with ICMJE requirements if the results were publishable. However, the recent nature of our sample is a possible explanation for our finding low rates of trial publication. Although we allowed at least 2 y after the study ended for publication, consistent with FDAAA legislation, rates were higher among those that ended longer ago. Nevertheless, publication rates reached only 60% among trials documented as having ended prior to 2004, an allowance of at least 4 y for trial publication. Importantly, all the trials included in our study had their registration updated to notify ClinicalTrials.gov that the trial had been completed.

Many studies have attempted to evaluate the extent of selective publication in the biomedical literature and found similarly low rates of publication [5],[12]–[24], although none have used, to our knowledge, as large and as broadly representative a registry as ClinicalTrials.gov, particularly with regards to condition studied and study location, with the exception of two recent studies focused on the publication of trials submitted to the FDA [1],[2]. Other evidence concerning selective publication is anecdotal, such as the absence of 6 mo of trial data from a key publication describing the efficacy of celecoxib [25],[26], the delay of publication for two early trials of rofecoxib until after the medication was withdrawn from the market [27]–[29], and the aforementioned studies of rosiglitazone [3], erythropoiesis-stimulating agents [4], and antidepressants [5].

However, as described, low publications rates were not limited to specific trial sponsors, suggesting that selective publication is an issue among trials sponsored by both industry and government and reinforcing the importance of registries like ClinicalTrials.gov for addressing this problem. Selective publication may occur for several reasons, although our study was not designed to evaluate its causes. If trial results put either investigators or the study's sponsor at financial risk, they may be delayed or suppressed [30]. In addition, if trial results contradict investigators' beliefs, providing unexpected support (or lack of support) for a particular clinical practice, they may not be submitted for publication [30]. This may be exacerbated by investigator reluctance to publish negative results given the need to highlight “positive, promising” findings for grant applications. Finally, researchers, reviewers, and editors have historically been more enthusiastic about positive or equivalence trials and less excited about negative trials [6]; accordingly, these latter trials are submitted and accepted for publication less often [14]–[16],[21],[31]. Suggestive of this, 70% of published manuscripts in our study from intervention-placebo or intervention-active trials were reported as positive, although we are unable to determine what proportion of the unpublished trials found positive results.

Although the FDAAA now requires reporting of trial results within 1 to 2 y after study completion within ClinicalTrials.gov, selective publication may not be fully remedied. The quality of the information provided for some data elements within ClinicalTrials.gov varied widely. It is not clear whether or how often the accuracy of the data is verified by the NLM, although those responsible for the conduct of the clinical trial are principally accountable for its quality and accuracy. Even though nearly all trials reported mandatory data elements, many entries were of poor quality and provided limited information, particularly the principal investigator/study contact. Reporting of optional data elements ranged widely and, similar to the mandatory data elements, many were of poor quality and provided limited information. As had been shown in prior research [7], only 66% of trials reported their primary outcome measure, and outcomes were often vague and poorly specific among those that did, making it difficult or impossible to detect outcome reporting bias. Given the documented presence of outcome reporting bias among trials studied in other settings [5],[12],[13], the potential impact of ClinicalTrials.gov on outcome reporting bias deserves further research. Just as significant progress has been made with regards to improved reporting of the study intervention (i.e., drug name) within ClinicalTrials.gov [32], progress can be made by mandating the registration of all information that is necessary for the public and profession to access and interpret trial results, including primary and secondary outcomes, study location, and enrollment, with clear field requirements to prevent vague reporting and improve data quality. Furthermore, we propose that either the NLM or another specified agency be given sufficient power of enforcement, including the capacity to assess fines or other penalties to sponsors or investigators who are not compliant with requirements.

One limitation of our study was that nearly half of the 10% subsample of trials among which we determined publication status did not report a trial end date, and those that did not were published at the lowest rates, preventing an assurance that all trials were allowed at least 2 y after study completion for trial publication. In addition, although all the trials included in our study had their registries updated to notify ClinicalTrials.gov that the trial had been completed, the date on which this specific notification was made was not available. This low rate of reporting of an optional data element (“study end date”) in itself suggests that reporting of information needed to comprehensively assess trial progress and completion must be required and verified. In addition, we cannot be certain of the relationship between not reporting trial end date and publication. Not reporting an end date may indicate that study officials had determined that the trial would not be submitted for publication and thus made minimal efforts to fully update the trial's registration within ClinicalTrials.gov, such as by providing the actual trial end date, outside of providing notification that the trial had been completed. Similarly, the low response rate among investigators surveyed about completed yet unpublished registered trials may indicate that the trials were not published and investigators were instead focused on current study efforts. Nevertheless, rates of publication were low among both trials that did and did not report end dates.

There are other limitations to our study. Relevant publications may not have been identified in our review, partly because we limited our study to MEDLINE and did not search other databases, such as EMBASE or research conference proceedings (abstracts). However, EMBASE is not publicly accessible, requiring a subscription for access. Moreover, research abstracts are often preliminary and rarely provide all relevant efficacy and safety findings. Our search for publications was extensive, involving two independent investigators using a systematic method to query MEDLINE. If we were unable to identify a trial publication, it is unlikely that others using PubMed to find results from a trial from ClinicalTrials.gov would be able to consistently do so. In addition, some studies may have been made publicly available elsewhere. In response to criticism about selective publication, several pharmaceutical companies and their US trade association (Pharmaceutical Research and Manufacturers of America) have established registries to report results of their clinical trials [33]–[36]. Although a useful step, these registries do not adequately address the issue of selective publication since the results are not subject to peer review and provide no assurance of complete reporting of efficacy and safety. Secondly, our sample size may have been too small for our analyses to have sufficient power to identify true differences in publication rates between trial subcategories, such as sponsorship or study purpose. Finally, many changes may have already been or will be made to ClinicalTrials.gov in response to addressing the new requirements enacted as part of the FDAAA. However, an important purpose of our study was to inform these efforts and future work will need to examine whether changes made the registry more effective.

The scientific community should be prioritizing the timely and accurate publication and dissemination of clinical trial results, regardless of the strength and direction of trial results. Current, up-to-date evidence is critical for clinicians, researchers, and patients, and late publication can impair and undermine evidence-based clinical practice almost as effectively as nonpublication. In addition, investigators have an obligation to ensure that the efforts of patients who volunteer as trial subjects are shared to advance science. Publication rates among completed trials registered within ClinicalTrials.gov were low, even among trials with at least 4 y since the study had ended. Critically, even among published trials, few reported the citation within ClinicalTrials.gov, a small but necessary step that should be required in order to make it easy for the public and the profession to have access to the trial results. The FDA needs a coordinated strategy for oversight and enforcement of the new requirements of the FDAAA, along with a commitment from industry, government, and all other trial sponsors, as well as the scientific community, to minimize selective publication of trials and ensure timely public and professional access to trial results.

## Supporting Information

Table S1 ClinicalTrials.gov mandatory and optional data elements for intervention trials.
(0.07 MB DOC)

## Abbreviations

CI confidence interval
FDAAA: FDA Amendments Act
RR: relative risk
NLM: US National Library of Medicine

## References

[1] Lee K, Bacchetti P, Sim I (2008). Publication of clinical trials supporting successful new drug applications: a literature analysis.. PLoS Med.

[2] Rising K, Bacchetti P, Bero LA (2008). Reporting bias in drug trials submitted to the Food and Drug Administration: review of publication and presentation.. PLoS Med.

[3] Nissen SE, Wolski K (2007). Effect of rosiglitazone on the risk of myocardial infarction and death from cardiovascular causes.. N Engl J Med.

[4] Bennett CL, Silver SM, Djulbegovic B, Samaras AT, Blau CA (2008). Venous thromboembolism and mortality associated with recombinant erythropoietin and darbepoetin administration for the treatment of cancer-associated anemia.. JAMA.

[5] Turner EH, Matthews AM, Linardatos E, Tell RA, Rosenthal R (2008). Selective publication of antidepressant trials and its influence on apparent efficacy.. N Engl J Med.

[6] DeAngelis CD, Drazen JM, Frizelle FA, Haug C, Hoey J (2004). Clinical trial registration: a statement from the International Committee of Medical Journal Editors.. JAMA.

[7] Zarin DA, Tse T, Ide NC (2005). Trial Registration at ClinicalTrials.gov between May and October 2005.. N Engl J Med.

[8] U. S. National Institutes of Health (2009). About ClinicalTrials.gov.. http://www.clinicaltrials.gov/ct2/info/about.

[9] U. S. National Institutes of Health (2009). ClinicalTrials.gov.. http://www.clinicaltrials.gov.

[10] U. S. National Institutes of Health (2009). Glossary of clinical trial terms.. http://www.clinicaltrials.gov/ct2/info/glossary.

[11] Lexchin J, Bero LA, Djulbegovic B, Clark O (2003). Pharmaceutical industry sponsorship and research outcome and quality: systematic review.. BMJ.

[12] Chan AW, Hrobjartsson A, Haahr MT, Gotzsche PC, Altman DG (2004). Empirical evidence for selective reporting of outcomes in randomized trials: comparison of protocols to published articles.. JAMA.

[13] Chan AW, Krleza-Jeric K, Schmid I, Altman DG (2004). Outcome reporting bias in randomized trials funded by the Canadian Institutes of Health Research.. CMAJ.

[14] Decullier E, Lheritier V, Chapuis F (2005). Fate of biomedical research protocols and publication bias in France: retrospective cohort study.. BMJ.

[15] Dickersin K, Min YI (1993). NIH clinical trials and publication bias..

[16] Dickersin K, Min YI, Meinert CL (1992). Factors influencing publication of research results. Follow-up of applications submitted to two institutional review boards.. JAMA.

[17] Easterbrook PJ, Berlin JA, Gopalan R, Matthews DR (1991). Publication bias in clinical research.. Lancet.

[18] Ioannidis JP (1998). Effect of the statistical significance of results on the time to completion and publication of randomized efficacy trials.. JAMA.

[19] Krzyzanowska MK, Pintilie M, Tannock IF (2003). Factors associated with failure to publish large randomized trials presented at an oncology meeting.. JAMA.

[20] Misakian AL, Bero LA (1998). Publication bias and research on passive smoking: comparison of published and unpublished studies.. JAMA.

[21] Stern JM, Simes RJ (1997). Publication bias: evidence of delayed publication in a cohort study of clinical research projects.. BMJ.

[22] von Elm E, Rollin A, Blumle A, Huwiler K, Witschi M (2008). Publication and non-publication of clinical trials: longitudinal study of applications submitted to a research ethics committee.. Swiss Med Wkly.

[23] Bardy AH (1998). Bias in reporting clinical trials.. Br J Clin Pharmacol.

[24] Dwan K, Altman DG, Arnaiz JA, Bloom J, Chan AW (2008). Systematic review of the empirical evidence of study publication bias and outcome reporting bias.. PLoS ONE.

[25] Silverstein FE, Faich G, Goldstein JL, Simon LS, Pincus T (2000). Gastrointestinal toxicity with celecoxib vs nonsteroidal anti-inflammatory drugs for osteoarthritis and rheumatoid arthritis: the CLASS study: a randomized controlled trial. Celecoxib Long-term Arthritis Safety Study.. JAMA.

[26] Juni P, Rutjes AW, Dieppe PA (2002). Are selective COX 2 inhibitors superior to traditional non steroidal anti-inflammatory drugs?. BMJ.

[27] Mukherjee D, Nissen SE, Topol EJ (2001). Risk of cardiovascular events associated with selective COX-2 inhibitors.. JAMA.

[28] Kivitz AJ, Greenwald MW, Cohen SB, Polis AB, Najarian DK (2004). Efficacy and safety of rofecoxib 12.5 mg versus nabumetone 1,000 mg in patients with osteoarthritis of the knee: a randomized controlled trial.. J Am Geriatr Soc.

[29] Weaver AL, Messner RP, Storms WW, Polis AB, Najarian DK (2006). Treatment of patients with osteoarthritis with rofecoxib compared with nabumetone.. J Clin Rheumatol.

[30] Blumenthal D, Campbell EG, Anderson MS, Causino N, Louis KS (1997). Withholding research results in academic life science. Evidence from a national survey of faculty.. JAMA.

[31] Dickersin K, Min YI (1993). Publication bias: the problem that won't go away.. Ann N Y Acad Sci.

[32] Zarin DA, Ide NC, Tse T, Harlan WR, West JC (2007). Issues in the registration of clinical trials.. JAMA.

[33] GlaxoSmithKline (2009). Clinical trial register.. http://ctr.gsk.co.uk/welcome.asp.

[34] Eli Lilly (2009). Clinical trial results.. http://www.lillytrials.com/results/results.html.

[35] Hoffman-La Roche (2009). Clinical trial results database.. http://www.roche-trials.com/results.html.

[36] Pharmaceu Research and Manufacturers of America (2009). http://www.clinicalstudyresults.org/home/.

