# Supplementary material for: Trial Publication after Registration in ClinicalTrials.Gov: A Cross-Sectional Analysis
Source: PLoS Med. 2009 Sep 8;6(9):e1000144. doi: 10.1371/journal.pmed.1000144 (PMC2728480; doi:10.1371/journal.pmed.1000144)
Supplement: Table S1 — ClinicalTrials.gov mandatory and optional data elements for intervention trials. (0.07 MB DOC) [file pmed.1000144.s001.doc]

**APPENDIX**

**Table S1:** ClinicalTrials.gov mandatory and optional data elements for intervention trials.

| **Data Element** | | | **Status** |
| --- | --- | --- | --- |
| Descriptive Information | | |  |
|  | Title | | Mandatory |
|  | Summary | | Mandatory |
|  | Primary purpose | | Optional |
|  | Design | | Mandatory |
|  | Phase | | Mandatory |
|  | Type | | Mandatory |
|  | Conditions or focus of study | | Mandatory |
|  | Intervention | | Mandatory |
|  | Start date | | Optional |
|  | Completion date | | Optional |
|  | Enrollment target number | | Optional |
|  | Primary outcome measure | | Optional |
|  | Secondary outcome measure | | Optional |
| Recruitment Information | | |  |
|  | Eligibility criteria | | Mandatory |
|  | Gender | | Mandatory |
|  | Minimum/Maximum age | | Mandatory |
|  | Accepts healthy volunteers? | | Optional |
|  | Overall recruitment status | | Mandatory |
| Location and Contact Information | | |  |
|  | Sponsor | | Mandatory |
|  | Responsible party | | Optional |
|  | Facility | | Mandatory |
|  | Study official/facility contact | | Mandatory |
|  | Central contact | | Mandatory |
| Administrative Information | | |  |
|  | Unique protocol identification | | Mandatory |
|  | Secondary identifications | | Optional |
|  | Investigational new drug/device? | | Mandatory |
|  | Record verification date | | Mandatory |
| Other Necessary Information | | |  |
|  | Applicable clinical trial?* | | Optional |
|  | FDA product status | | Optional |
|  | Institutional review board approval? | | Mandatory |
|  | Oversight authorities | | Mandatory |
|  | NCT number | | Assigned by ClinicalTrials.gov |
|  | First received date | | Assigned by ClinicalTrials.gov |
|  | References | |  |
|  |  | MEDLINE identifier | Optional |
|  |  | Citation | Optional |
|  | Links | | Optional |

FDA=Food and Drug Administration; NCT=ClinicalTrials.gov registration prefix.

* Asks whether study is a clinical trial of an FDA regulated intervention, a section 801 clinical trial, or a delayed posting.
